# Supplementary material for: Exercise Training Differentially Affects Skeletal Muscle Mitochondria in Rats with Inherited High or Low Exercise Capacity
Source: Cells. 2024 Feb 24;13(5):393. doi: 10.3390/cells13050393 (PMC10931189; doi:10.3390/cells13050393)
Supplement: Supplementary file 1 [file cells-13-00393-s001.zip › cells-2828720-supplementary.pdf]

Table S1: Additional echocardiographic parameters of sedentary and trained rats with high (HCR) or low (LCR) intrinsic exercise capacity

	HCR (n=8)	HCR ex (n=6)	LCR (n=8)	LCR ex (n=10)	G	E	I
LVAWd [mm]	1.65 ± 0.06	1.78 ± 0.09	1.68 ± 0.07	1.83 ± 0.08	ns	ns	ns
LVAWs [mm]	2.64 ± 0.06	2.84 ± 0.15	2.95 ± 0.07†	3.00 ± 0.08	*	ns	ns
LVPWd [mm]	1.49 ± 0.06	1.46 ± 0.10	1.67 ± 0.09	1.54 ± 0.05	ns	ns	ns
LVPWs [mm]	2.24 ± 0.10	2.19 ± 0.11	2.69 ± 0.12††	2.49 ± 0.11	**	ns	ns
MV VTI [mm/s]	539 ± 29	379 ± 7**	485 ± 50	404 ± 10	ns	***	ns
MV E [mm/s]	805 ± 28	731 ± 24	754 ± 60	786 ± 17	ns	ns	ns
MV A [mm/s]	538 ± 41	434 ± 26	476 ± 49	478 ± 16	ns	ns	ns
E\` [mm/s]	57.4 ± 2.4	48.5 ± 1.3*	52.3 ± 2.8	46.4 ± 2.2	ns	**	ns
A\` [mm/s]	43.2 ± 3.8	33.3 ± 2.6	37.0 ± 3.9	34.7 ± 1.3	ns	ns	ns
S\` [mm/s]	36.4 ± 1.8	34.3 ± 3.2	36.1 ± 2.5	33.3 ± 1.3	ns	ns	ns
EF [%]	71.1 ± 2.3	70.5 ± 3.4	83.1 ± 2.3††	77.5 ± 2.0	***	ns	ns
LVMI [g/m^2]	3.44 ± 0.24	3.94 ± 0.25	2.98 ± 0.11	3.33 ± 0.21	*	ns	ns
MV E/A	1.56 ± 0.14	1.72 ± 0.15	1.68 ± 0.15	1.66 ± 0.05	ns	ns	ns
E\`/A\`	1.38 ± 0.10	1.49 ± 0.07	1.54 ± 0.19	1.34 ± 0.05	ns	ns	ns

Data are mean ± SEM. Ex – exercise trained, G - genetically determined aerobic exercise capacity; E – exercise training; I – interaction; LVAWd – left ventricular anterior wall thickness in diastole, LVAWs – left ventricular anterior wall thickness in systole, LVPWd – left ventricular posterior wall thickness in diastole, LVPWs – left ventricular posterior wall thickness in systole, MV - mitral valve, VTI – velocity time integral, E – early, passive filling of left ventricle (E-wave), MV A – late, active filling of left ventricle (A-wave), E\`- wall velocity during E, A\` - velocity during A, S\` - systolic velocity, EF – ejection fraction, LVMI – left ventricular mass index, ns – non-significant, n = 6-10 for echocardiography, * p<0.05, ** p<0.01, *** p<0.001 for G, E, I or for exercise training; † p<0.05, †† p<0.01 for intrinsic exercise capacity

Table S2: Maximal respiratory capacity of interfibrillar mitochondria of sedentary and trained rats with high (HCR) or low (LCR) intrinsic exercise capacity

		HCR	HCR ex	LCR	LCR ex	G	E	I
**Glutamate**	State 4	30.6 ± 3.3	47.5 ± 10.2	36.4 ± 3.5	43.9 ± 6.8	ns	ns	ns
HCR n=4, HCR ex n=8	RCI	5.21 ± 0.60	4.11 ± 0.34	3.55 ± 0.41†	2.33 ± 0.13††	***	**	ns
LCR n=4-5, LCR ex n=5-6	DNP	405 ± 29	532 ± 105	298 ± 10	321 ± 37	ns	ns	ns
	ADP/O	1.57 ± 0.09	2.47 ± 0.32*	1.46 ± 0.08	1.65 ± 0.05†	ns	*	ns
**Glutamate/malate**	State 4	400 ± 41	63.0 ± 9.1	45.1 ± 3.4	56.7 ± 7.3	ns	ns	ns
HCR n=11-12, HCR ex n=10-11	RCI	6.94 ± 0.22	5.89 ± 0.30*	5.73 ± 0.38††	4.05 ± 0.45**††	***	***	ns
LCR n=12, LCR ex n=7	DNP	706 ± 57	672 ± 94	412 ± 43††	372 ± 29††	***	ns	ns
	ADP/O	1.59 ± 0.11	2.13 ± 0.18*	1.45 ± 0.06	2.36 ± 0.41**	ns	***	ns
**Pyruvate/malate**	State 4	68.0 ± 9.2	82.5 ± 14.8	38.8 ± 4.8†	50.6 ± 5.6†	**	ns	ns
HCR n=11-12, HCR ex n=10-11	RCI	4.30 ± 0.23	3.42 ± 0.20**	4.27 ± 0.19	3.03 ± 0.19***	ns	***	ns
LCR n=11-12, LCR ex n=7	DNP	155 ± 19	152 ± 23	96.2 ± 9.7†	101 ± 11	**	ns	ns
	ADP/O	1.66 ± 0.06	2.08 ± 0.16*	1.58 ± 0.09	2.05 ± 0.20*	ns	***	ns
**PCoA/car/malate**	State 4	41.4 ± 5.9	62.1 ± 11.1	39.1 ± 2.4	57.4 ± 6.7	ns	*	ns
HCR n=4-5, HCR ex n=6-7	RCI	3.74 ± 0.38	3.80 ± 0.44	2.99 ± 0.31	2.59 ± 0.15†	*	ns	ns
LCR n=5, LCR ex n=7	DNP	139 ± 4	266 ± 83	121 ± 14	163 ± 25	ns	ns	ns
	ADP/O	1.43 ± 0.12	1.82 ± 0.15	1.35 ± 0.14	1.78 ± 0.30	ns	ns	ns
**Pc/malate**	State 4	54.03 ± 5.35	64.3 ± 10.3	44.1 ± 1.9	52.9 ± 5.4	ns	ns	ns
HCR n=10-11, HCR ex n=10-11	RCI	3.87 ± 0.21	4.55 ± 0.41	3.42 ± 0.21	3.10 ± 0.17††	**	ns	ns
LCR n=8, LCR ex n=7	DNP	244 ± 33	343 ± 64	144 ± 13	183 ± 22†	**	ns	ns
	ADP/O	1.37 ± 0.07	1.74 ± 0.10	1.38 ± 0.08	1.89 ± 0.37	ns	*	ns
**Succinate/rotenone**	State 4	102.1 ± 7.8	110.7 ± 16.4	74.5 ± 7.6	82.0 ± 12.8	*	ns	ns
HCR n=12-13, HCR ex n=8-10	RCI	3.86 ± 0.07	3.69 ± 0.11	3.24 ± 0.19††	3.36 ± 0.16	**	ns	ns
LCR n=10, LCR ex n=7	DNP	384 ± 36	392 ± 50	266 ± 38†	313 ± 46	*	ns	ns
	ADP/O	0.87 ± 0.06	0.94 ± 0.08	0.73 ± 0.08	1.29 ± 0.16***†	ns	**	*
**DHQ/rotenone**	State 4	109.8 ± 16.0	204.4 ± 29.1*	95.9 ± 8.8	123.5 ± 26.7†	ns	*	ns
HCR n=4-5, HCR ex n=9-11	RCI	3.65 ± 0.11	3.58 ± 0.12	3.86 ± 0.29	3.74 ± 0.22	ns	ns	ns
LCR n=4-5, LCR ex n=5-7	DNP	546 ± 69	1084 ± 152	535 ± 40	527 ± 123	ns	ns	ns
	ADP/O	0.77 ± 0.06	0.89 ± 0.07	0.69 ± 0.04	1.10 ± 0.15*	ns	*	ns

Data are mean ± SEM. Ex – exercise trained, G - genetically determined aerobic exercise capacity; E – exercise training; I – interaction; PCoA – palmitoyl coenzyme A, car - carnitine, Pc – palmitoylcarnitine, DHQ - durohydroquinone, RCI – ratio between state 3 and state 4, DNP – 2,4-dinitrophenol, ADP/O – ratio of ADP phosphorylated to atoms of oxygen consumed, ns – non-significant, n = 5-12, * $p<0.05$, ** $p<0.01$, *** $p<0.001$ for G, E, I or for exercise training; † $p<0.05$, †† $p<0.01$ for intrinsic exercise capacity

Table S3: Maximal respiratory capacity of subsarcolemmal mitochondria of sedentary and trained rats with high (HCR) or low (LCR) intrinsic exercise capacity

		HCR	HCR ex	LCR	LCR ex	G	E	I
**Glutamate**	State 4	120 ± 19	113.2 ± 20.8	152 ± 26	184 ± 66	ns	ns	ns
HCR n=5, HCR ex n=8	RCI	2.88 ± 0.13	2.88 ± 0.33	2.59 ± 0.37	2.17 ± 0.18	ns	ns	ns
LCR n=5, LCR ex n=6	DNP	578 ± 81	615 ± 120	565 ± 139	658 ± 225	ns	ns	ns
	ADP/O	1.30 ± 0.15	1.53 ± 0.14	1.11 ± 0.21	1.57 ± 0.18	ns	ns	ns
**Glutamate/malate**	State 4	109 ± 6	137 ± 26	147 ± 21	229 ± 83	ns	ns	ns
HCR n=10-12, HCR ex n=11	RCI	4.12 ± 0.19	4.16 ± 0.42	2.43 ± 0.16†††	2.20 ± 0.16†††	***	ns	ns
LCR n=11, LCR ex n=6	DNP	640 ± 50	723 ± 117	541 ± 97	828 ± 349	ns	ns	ns
	ADP/O	1.35 ± 0.10	1.73 ± 0.12	1.08 ± 0.08	1.81 ± 0.64*	ns	*	ns
**Pyruvate/malate**	State 4	134 ± 10	172 ± 30	145 ± 23	232 ± 91	ns	ns	ns
HCR n=10-12, HCR ex n=10-11	RCI	2.82 ± 0.18	2.45 ± 0.13	2.25 ± 0.11††	1.91 ± 0.13†	***	*	ns
LCR n=11, LCR ex n=6	DNP	170 ± 8	201 ± 33	152 ± 29	247 ± 88	ns	ns	ns
	ADP/O	1.41 ± 0.10	1.66 ± 0.15	1.03 ± 0.09	1.59 ± 0.31*	ns	*	ns
**PCoA/car/malate**	State 4	153 ± 31	144 ± 25	155 ± 33	238 ± 119	ns	ns	ns
HCR n=5, HCR ex n=6-7	RCI	2.33 ± 0.30	2.44 ± 0.27	2.56 ± 0.34	2.00 ± 0.17	ns	ns	ns
LCR n=5, LCR ex n=4-5	DNP	289 ± 63	234 ± 34	293 ± 62	389 ± 199	ns	ns	ns
	ADP/O	1.29 ± 0.17	1.45 ± 0.09	1.13 ± 0.16	1.02 ± 0.13†	*	ns	ns
**Pc/malate**	State 4	107 ± 7	122 ± 17	186 ± 22	272 ± 127†	**	ns	ns
HCR n=8-9, HCR ex n=10-11	RCI	3.14 ± 0.24	2.90 ± 0.25	1.88 ± 0.17††	2.12 ± 0.17	***	ns	ns
LCR n=7, LCR ex n=3-4	DNP	285 ± 20	288 ± 42	271 ± 44	475 ± 185	ns	ns	ns
	ADP/O	1.28 ± 0.09	1.34 ± 0.11	0.97 ± 0.08†	1.05 ± 0.12	*	ns	ns
**Succinate/rotenone**	State 4	143 ± 14	190 ± 32	175 ± 26	244 ± 85	ns	ns	ns
HCR n=10-11, HCR ex n=10-11	RCI	3.11 ± 0.18	2.83 ± 0.14	2.07 ± 0.15†††	2.70 ± 0.27*	**	ns	*
LCR n=8-9, LCR ex n=6	DNP	460 ± 65	423 ± 56	390 ± 68	644 ± 230	ns	ns	ns
	ADP/O	0.78 ± 0.04	0.85 ± 0.06*	0.57 ± 0.08†	0.86 ± 0.11	ns	*	ns
**DHQ/rotenone**	State 4	310 ± 78	297 ± 54	267 ± 48	292 ± 75	ns	ns	ns
HCR n=5, HCR ex n=10-11	RCI	2.87 ± 0.33	3.20 ± 0.12	2.65 ± 0.19	2.79 ± 0.33	ns	ns	ns
LCR n=5, LCR ex n=6	DNP	1096 ± 215	1287 ± 248	923 ± 205	1065 ± 299	ns	ns	ns
	ADP/O	0.69 ± 0.07	0.83 ± 0.07	0.50 ± 0.05	0.67 ± 0.17	ns	ns	ns

Data are mean ± SEM. Ex – exercise trained, G - genetically determined aerobic exercise capacity; E – exercise training; I – interaction; PCoA – palmitoyl coenzyme A, car - carnitine, Pc – palmitoylcarnitine, DHQ - durohydroquinone, RCI – ratio between state 3 and state 4, DNP – 2,4-dinitrophenol, ADP/O – ratio of ADP phosphorylated to atoms of oxygen consumed, ns – non-significant, n = 3-12, * $p<0.05$, ** $p<0.01$, *** $p<0.001$ for G, E, I or for exercise training; † $p<0.05$, †† $p<0.01$, ††† $p<0.001$ for intrinsic exercise capacity
